# UniProtExtractR: an app and R package for easily extracting protein-specific UniProtKB information and fine-tuning organelle resolution

**DOI:** 10.1093/bioadv/vbad157

**Published:** 2023-10-31

**Authors:** Alexandra Panov, J Wade Harper

**Affiliations:** Department of Cell Biology, Harvard Medical School, Boston, MA 02115, United States; Aligning Science Across Parkinson’s (ASAP) Collaborative Research Network, Chevy Chase, MD 20815, United States; Department of Cell Biology, Harvard Medical School, Boston, MA 02115, United States; Aligning Science Across Parkinson’s (ASAP) Collaborative Research Network, Chevy Chase, MD 20815, United States

## Abstract

**Summary:**

UniProtKB is a publicly accessible database of annotated protein features for numerous organisms; however, globally extracting protein entry information for data visualization and categorization can be challenging. While the UniProtKB entry syntax maintains database consistency, it simultaneously obscures key terms within long character strings. To increase accessibility, UniProtExtractR is both an app and R package that extracts desired information across nine UniProtKB categories: DNA binding, Pathway, Transmembrane, Signal peptide, Protein families, Domain [FT], Motif, Involvement in disease, and Subcellular location [CC]. The app features interactive frequency tables that globally summarize both the original UniProtKB input query as well as the extracted/changed entry values. Moreover, UniProtExtractR includes a tractable mapping algorithm to define custom organelle-level resolution. UniProtExtractR exists as a freely accessible Shiny app that requires no coding experience as well as R package, the code of which is entirely open source.

**Availability and implementation:**

UniProtExtractR source code and user manual, including example files and troubleshooting, is available at https://github.com/alex-bio/UniProtExtractR. The Shiny app is hosted at https://harperlab.connect.hms.harvard.edu/uniprotextractR.

## 1 Introduction

In the age of -omics level datasets in biomedical research, data visualization and categorization are critical. The Universal Protein Resource (UniProt) has maintained a high quality, publicly accessible, and expertly curated database for integrated protein information called UniProt Knowledgebase (UniProtKB), which currently houses over 227 million protein sequences across all domains of life (The UniProt Consortium 2007, 2023a). Features within this database include protein families and domains, post-translations modifications (PTMs), disease-specific variants, structural information, among many others. Information from UniProtKB can be accessed by manually downloading from their website, https://www.uniprot.org, or by querying from one of several UniProt application programming interfaces (APIs). However, many features are maintained as character strings following a rigid syntax that prove difficult for data analysis; as an example, subcellular location takes the following character string format:

“SUBCELLULAR LOCATION:(( *Molecule*:)?(*Location*\.)+)?( Note=*Free_text* (*Flag*)?\.)?”

(The UniProt Consortium, 2023b). While the chosen syntax allows for consistency across all entries, it remains inaccessible for scalable, high-throughput data analysis. In addition, complex features of protein properties, such as multiple protein localization descriptors and inconsistencies across annotated data, limit the resource usefulness for analyses that rely on biologically precise assignments.

To address the issue of inaccessibility and extractability, we built a Shiny app and R package called UniProtExtractR. This tool has three primary functions; (i) it extracts desired information from the syntactically structured character strings across nine UniProtKB categories: DNA binding, Pathway, Transmembrane, Signal peptide, Protein families, Domain [FT], Motif, Involvement in disease, and Subcellular location [CC]; (ii) a dashboard of frequency tables summarizes the input UniProtKB query; and (iii) it includes a tunable mapping feature for assigning and extracting user-customized organelle localization descriptors at the desired level of resolution.

## 2 Features

This tool was designed with ease of access as the highest priority. In order to make the extraction tool as accessible as possible, we built a Shiny app that can be found at https://harperlab.connect.hms.harvard.edu/uniprotextractR. The R package can be found on GitHub at https://github.com/alex-bio/UniProtExtractR alongside a thorough manual.

**Figure 1.**
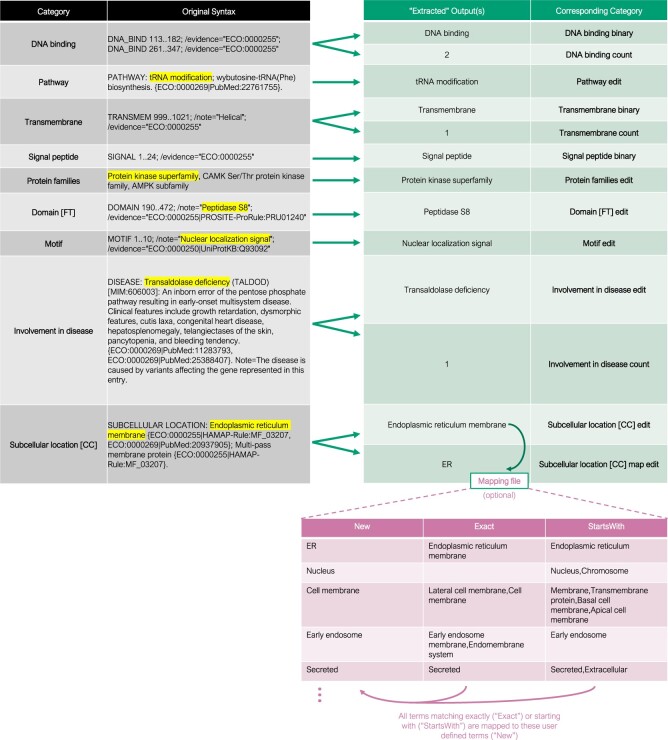
Summary of input (gray, top left) UniProtKB categories (The UniProt Consortium, 2023a), UniProtExtractR output (bluish green, top right), and example optional mapping file (pink, bottom). The highlighted terms from the gray input categories are extracted as the bluish green new categories. Some input categories are split into binary or count categories, which indicate if a protein entry contains that property and how many, respectively. All added categories end with “binary,” “count,” or “edit.” For Subcellular location [CC] edit, the optional mapping file (pink, bottom) redefines specific terms (“Exact” or “StartsWith”) to customizable terms (“New”) for user-defined organelle resolution, adding a final category, Subcellular location [CC] map edit.

In brief, UniProtKB may be directly queried (whole proteome or custom entries) or any UniProtKB query can be uploaded into the Shiny app or R package function as a .TSV file or R dataframe, respectively. We recommend directly querying UniProtKB in the app to keep the process as streamlined as possible; this feature leverages the UniProt REST API using the UniProt.ws package (Carlson and Ramos, 2023). To directly download from the UniProtKB webpage (https://uniprot.org), a user may search any custom query and then click the “Download” button and select .TSV format. The Shiny app at the link above has a 5 GB upload size limit, while the R package has none; because the Shiny app code is also on GitHub, a user can change the 5 GB limit if running locally. UniProtExtractR searches for the presence of any of the aforementioned nine categories; for any category identified (Fig. 1, gray, top left), a new column(s) will be added to the original UniProtKB query file immediately adjacent to the category (Fig. 1, bluish green, top right). The core utilities of UniProtExtractR leverage regular expression for extracting information. For example, if a UniProtKB query includes a column for Protein families, UniProtExtractR will add a syntactically edited or “extracted” Protein families column immediately after the original Protein families column. An example of UniProtKB query file directly exported from UniProtKB is on GitHub to demonstrate input file structure and column names. After running, both the original and modified columns will be present in the downloadable output file for user comparison. For the categories DNA binding, Transmembrane, and Signal peptide, a binary category is created indicating whether a protein entry has that feature or not. In addition, for DNA binding, Transmembrane, and Involvement in disease categories specifically, UniProtExtractR adds a numeric column counting the number of entities present per entry. Examples of how the remaining categories are extracted is shown in Fig. 1. Added categories ending with “binary” are categorical, indicating whether or not the UniProtKB entry contains that property. “Count” indicates how many entities are present per entry, and “edit” indicates that category has been extracted from UniProtKB syntax. For most categories, if there are multiple associations present, only the first listed value is extracted, which UniProt has assigned as the highest priority value; however, if a user desires, there are documented regions in the R package code that allow for expansion to multiple terms per entry.

For the Shiny app, any user can directly query UniProtKB or upload their UniProtKB query of interest into the app and launch “Run ExtractR.” Depending on the query size, running UniProtExtractR can take several minutes. The edited result file will be available for download upon completion. A main feature of the app is a sortable frequency table that appears upon run completion with a drop-down menu to show the distinct values in each column, both originally uploaded and newly added. In the era of large-scale data, this table paints a broad overview of the input dataset for any UniProtKB query without requiring any coding experience. The frequency table also counts how many blank values exist in each category to inform category coverage quality. As one example, the frequency table presents the counts of proteins with different numbers of transmembrane domains. Moreover, the sortable table will help summarize the extracted changes to the user, especially Subcellular location [CC].

One category of great interest for research in organelle turnover, cellular trafficking, or spatial-omics is Subcellular location [CC]. However, this category has a myriad of terms ranging from extremely specific organellar compartments to broadly organelle-wide. UniProtExtractR was designed with a key tractable feature, a mapping file (Fig. 1, pink, bottom), to configure the exact desired resolution of protein localizations. The mapping file contains three columns: “New,” “Exact,” and “StartsWith.” An example mapping file is available on GitHub. For every extracted Subcellular location [CC] per entry in the UniProtKB query, any terms that match exactly to the terms in “Exact” or that start with the phrases in “StartsWith” will be swapped for the value in “New” in a row-wise fashion. “Exact” and “StartsWith” may have many listed phrases, allowing for a user to define the limits of resolution across each cellular compartment. For example, a user may redefine an entire organism proteome with organelle-wide terms (e.g. “Nucleus,” “Cytoplasm”) for all organelles, or for all organelles except for one or two that retain high compartment specificity (e.g. “Mitochondrial inner membrane,” “Mitochondrial matrix”). We envision this feature to be particularly useful in navigating complexities and inconsistencies of protein localization from data in the literature as annotated in UniProtKB.

## 3 Conclusions

UniProtExtractR fills an access gap between the invaluable resource UniProtKB and researchers, particularly for data analysis purposes. This app and R package allow for categorization across many informative UniProtKB categories for any organism and any dataset, greatly expanding data visualization and modeling possibilities. Importantly, the app is accessible to all users independent of computer programming experience. In addition, this tool allows users to define protein localization resolution to best serve each experiment or project, which will greatly improve systematic exploration and discoverability within large datasets.
